# Bioinformatics-based discovery of biomarkers and immunoinflammatory infiltrates in hip fractures complicating deep vein thrombosis: A STROBE

**DOI:** 10.1097/MD.0000000000040809

**Published:** 2024-12-27

**Authors:** Zhijiang Fu, Chao Song, Yongliang Mei, Daqian Zhou, Yang Zhou, Jingwen Chen, Fei Liu, Qing Shang, Zongchao Liu

**Affiliations:** a Department of Orthopedics and Traumatology (Trauma and Bone-setting), The Affiliated Traditional Chinese Medicine Hospital, Southwest Medical University, Luzhou, Sichuan Province, China; b Department of Orthopedics, RuiKang Hospital Affiliated to Guangxi University of Chinese Medicine, Nanning, Guangxi, China; c Department of Orthopedics, People’s Hospital of Fushun County, Zigong, Sichuan Province, China; d Luzhou Longmatan District People’s Hospital, Luzhou, Sichuan Province, China.

**Keywords:** biomarkers, deep vein thrombosis, hip fracture, immune infiltration, vascularization

## Abstract

Deep vein thrombosis due to hip fracture is a normally concomitant symptom when fracture arrival at middle-aged and olderly people, but its molecular mechanism is still not well explained. We hypothesized that there are several key biomarkers and associated signaling pathways that could predict deep vein thrombosis, our goal was to employ bioinformatics to find important biomarkers of deep vein thrombosis and the results of immune infiltration. From the GEO-NCBI database, venous thrombosis expression profiles were chosen, and hip fracture and venous thrombosis gene datasets were gathered from a comprehensive database that can be searched for human genes, which is called GeneCards. Building networks between proteins with the STRING web application, and Kyoto Encyclopedia of Genes and Genomes (KEGG) and Gene Ontology (GO), 2 bioinformatic analytic tools, were used to conduct functional enrichment investigations. CIBERSORT was used to assess genetic data on the potential location of immune cells in venous thrombosis. Ultimately, 38 distinct genes from the first round and 10 crucial genes from the second round. GO and KEGG analyses showed that the intersecting distinct targets were enriched in routes of signaling mediated by chemokines, immune responses, and Inflammatory reactions were all involved, with the Jak-STAT and HIF-1 signaling pathways being the most significant. Immune cell infiltration analysis showed that immune inflammatory responses regulated by macrophages, and B cell, T cell all play a significant role in venous thrombosis. In conclusion, HIF-1, Jak-STAT signaling pathway, and mangy hub genes regulating inflammatory factors, and immune cells. They have a significant part in the venous thrombosis disease process.

## 1. Introduction

Hip fracture (HF) is a typical kind of fracture in middle-aged and elderly people, and as people age increase, the prevalence of HF rises.^[1]^ One of the most frequent side effects of hip fractures and surgery is venous thromboembolism (VTE), whereas the fourth most prevalent cause of mortality for patients with hip fractures is pulmonary embolism (PE).^[2]^ PE is mostly caused by deep vein thrombosis (DVT). Therefore, for HF in middle-aged and elderly people, treating fractures is crucial, but so is preventing DVT from developing.^[3]^ In the field of orthopedic trauma, doctors have always emphasized the early therapy for elderly patients with hip fractures, and have formulated the principle of “48-hour green channel for hip fracture in the elderly” to ensure that patients “pass through the hospital without delay” as quickly as possible, which is also in line with the concept of “rapid rehabilitation” advocated by the international community. Previous studies of hip fracture-related VTE have focused on the perioperative period, with fewer reports on the proportion of VTE during that time between injury and hospital admission.^[4]^ In fact, in the prehospital phase, injured person who suffers a hip fracture is at a significantly increased risk of adverse events due to their inability to receive VTE-related care and preventive measures in a timely manner. Studies have reported delayed hospitalization as a major cause of hip fractures. In the absence of any prophylaxis, the percentage of DVT in hip fracture can increase to 40% to 60%.^[5–7]^ Although advances have been made in the pathogenesis of VTE, the in-depth molecular mechanisms are far from being elucidated. Identifying factors that increase the chance of developing VTE in hip fractures remains an important health issue.

DVT is among the most frequent patient consequences hospitalized for major orthopedic surgery and an important complication of hip fracture in the elderly.^[5]^ Due to the insidious onset of DVT, clinical symptoms and signs of individual differences, leakage of diagnosis, misdiagnosis rate is high, often referred to as the “silent killer.”^[6]^ Although DVT can be predicted on the basis of specific clinical indicators, the predictive efficacy of a single indicator has limitations. For example, D-dimer has high diagnostic sensitivity but low specificity and can be elevated in trauma, surgery, myocardial infarction, malignancy, and inflammation.^[7]^ Related studies also didn’t find significant divergence in statistics in D-dimer levels between the thrombus and control groups, perhaps related to the fracture trauma itself, the inflammatory immune response provoked by the trauma, and other factors, such that D-dimer levels were elevated in both the thrombus and control groups.^[8]^ Blood stasis, vascular injury, and hypercoagulable state of blood are the 3 elements of DVT formation. Firstly, One of the high-risk factors for DVT is HF itself. Secondly, patients’ prolonged braking after fracture can lead to a decrease in the function of muscle-vessel pumps, coupled with the fact that most of them are elderly people, whose blood vessel wall elasticity decreases, leading to a slowing down of venous blood flow. Finally, the fracture itself can also damage the endothelium, release inflammatory mediators, form a chain reaction of inflammation, and directly or indirectly promote the formation of hypercoagulable state.^[9]^ Inflammatory molecules and immune cells are now recognized as central factors in thrombosis. Risk factors for venous thromboembolism in many clinical situations, including inflammatory bowel disease, systemic lupus erythematosus, obesity, surgery, cancer, and acute and chronic infections. It is characterized by dysregulation of the immune network intersecting with coagulation.^[9–11]^ It has been found that the onset of DVT is facilitated by the release of Weibel–Palade vesicles from endothelial cells and local endothelial activation. DVT is caused by a local inflammatory response that depends on mast cell activation and degranulation. Because they provide a scaffold for platelets and erythrocytes to adhere to, as well as because they encourage the production of thrombin and the deposition of fibrin, neutrophil cells are crucial in DVT.^[12]^ In conclusion, DVT is significantly influenced by the immunological inflammatory response, but the specific molecular mechanisms need to be further discovered. Thus, we hypothesized that there are several biomarkers and signaling pathways that can be predictors of DVT formation, and that simultaneously detecting these indicator changes in advance can have a good prognosis for DVT.

To discover whether our outcomes genes regulating immune cells and immune inflammation are present in the genes of patients with lower extremity venous thrombosis complicated by hip fracture, we performed relevant bioinformatics analyses. In this study, from the GEO-NCBI database, VTE expression profiles GSE19151 were chosen. HF, VTE related disease targets were downloaded from the GeneCards database. This study sought to identify useful biomarkers for the diagnosis and management of lower extremity VTE as well as the molecular mechanisms underlying the condition’s complications in HF.

## 2. Material and methods

### 2.1. Chip data collection and differentiated presentation

From the GEO-NCBI database, VTE expression profiles GSE19151 were chosen. Chip data from GSE19151 studied 70 patients with VTE previously treated with warfarin, and 63 healthy person. GSE19151 dataset sequenced on GPL571, all human-derived.^[13]^ Finding the genes that differ between VTE and healthy people through differential gene presentation with Sangerbox 3.0’s “limma” tool in order to investigate the effect of relevant gene expression levels on venous thromboembolism.^[14]^ The results of genes with differential expression were displayed using heat maps and volcanoes.

### 2.2. Weighted gene co-expression network analysis

The gene co-expression network of GSE19151 was built using the Sangerbox 3.0 weighted gene co-expression network analysis (WGCNA) software. The 2 sections of WGCNA’s methodology are expression clustering analysis and phenotypic association, which primarily consists of 4 steps: module-trait association, co-expression network, gene module identification, and correlation coefficient computation between genes. Using weighted expression correlations to build gene co-expression networks. Identify gene sets: based on weighted correlations, do hierarchical clustering analysis and trim the clustering results according to set criteria to produce various gene modules, represented by branches of the clustering tree and different colors. When phenotypic data is available, trait-related modules are found by calculating the correlation between gene modules and phenotypes. Examine how models relate to one another and observe the system-level interoperability network of various models. Choose driver genes of interest from important models, or make assumptions about the role of unknown genes by looking at the role of known genes in the model. Plot correlations after determining the TOM matrix.^[15]^

### 2.3. Access to DVT-related disease datasets

In order to further investigate the targets and effects of VTE after fracture, the keywords “hip fracture, deep vein thrombosis” were used. Search GeneCards, for disease target proteins.^[16,17]^ The DVT illness gene collection was obtained by merging and de-duplicating the search results from several databases.

### 2.4. Deep vein thrombosis biomarker identification

We obtained the intersection gene set by intersecting the DVT illness gene dataset with the Gene Symbol of the GEO integrated dataset. This allowed us to precisely identify the important biomarkers involved in the DVT process. The key genes involved in the DVT process are those in this intersecting gene set. Understanding cellular physiology in both healthy and diseased conditions depends on an understanding of protein–protein interactions, which underpin the majority of biological events in live cells. Protein-protein interaction (PPI) network studies were conducted in this study on the set of intersecting genes acquired for the species limited to “Homo sapiens” with a confidence value >0.4 using the STRING database. Cytoscape software (version 3.9.1) was used to build the PPI networks.^[18]^ Furthermore, important gene sets were obtained using the CytoHubba algorithm, a plug-in for the Cytoscape software.^[19]^ The CytoHubba method was used to screen the critical gene set in order to further identify the genes involved in the DVT process, and the pivotal genes with important functions were ultimately identified. To extract the differential gene expression between DVT samples and normal samples, the crucial genes were entered into the GEO integrated dataset lookup. Box-and-line plots were used to illustrate the results of the differential gene expression. Without cross-validation, the sample size this time is in line with that of data GSE19151.

### 2.5. Combining research on gene-based biological processes

Limit the species to “H.sapiens” after importing the hub gene set into Sangerbox 3.0 BioCloud and choosing Enrichment Analysis in the Tool Center. In the common parameters, type the hub gene set’s gene symbol and hit submit. Ultimately, we acquired hub gene Kyoto Encyclopedia of Genes and Genomes (KEGG) database pathway analysis and Gene Ontology (GO) enrichment analysis, and the outcomes are displayed in various diagrams.^[20,21]^

### 2.6. Recognition of disease-immune infiltrating cells

Immunological cells, inflammatory cells, fibroblasts, mesenchymal cells, and various cytokines and chemokines typically make up the immunological microenvironment.^[22]^ Cibersort was initially published in Nature Method in 2015 and is presently the most mentioned tool for the investigation of immune cell infiltration estimation. Based on the ideas of linear support vector regression (LVR), cibersort is a method for deconvolution of expression matrices of human immune cell subtypes. CIBERSORT It works better than other techniques for deconvolution analysis of unknown mixes and expression matrices with similar cell types, and it can be used for both microarray and sequencing expression matrices.^[23]^ The immune cell types of patients with varying immunization patterns in the dataset were determined using the CIBERSORT algorithm in the Sangerbox 3.0 software. The immune cell differences between patients with varying immunization patterns were displayed using a difference box-and-line plot. Additionally, we practically conducted Gene Set Enrichment study (GESA) on GSE19151 to further corroborate the findings of immune infiltration study and enrichment analysis.^[24]^

### 2.7. Statistical analysis

For statistical analysis, SPSS 20.0 software was utilized, and the data were represented as *x* ± *s*. A 1-way ANOVA was used to compare the means between numerous groups, the independent samples t-test was used to compare the means between 2 groups, and the LSD test was used to compare the means between 2 groups. Every statistical analysis result was deemed significant when *P* was <.05.

## 3. Results

### 3.1. Results that are differentiated depending on microarray data

**Figure 1.**
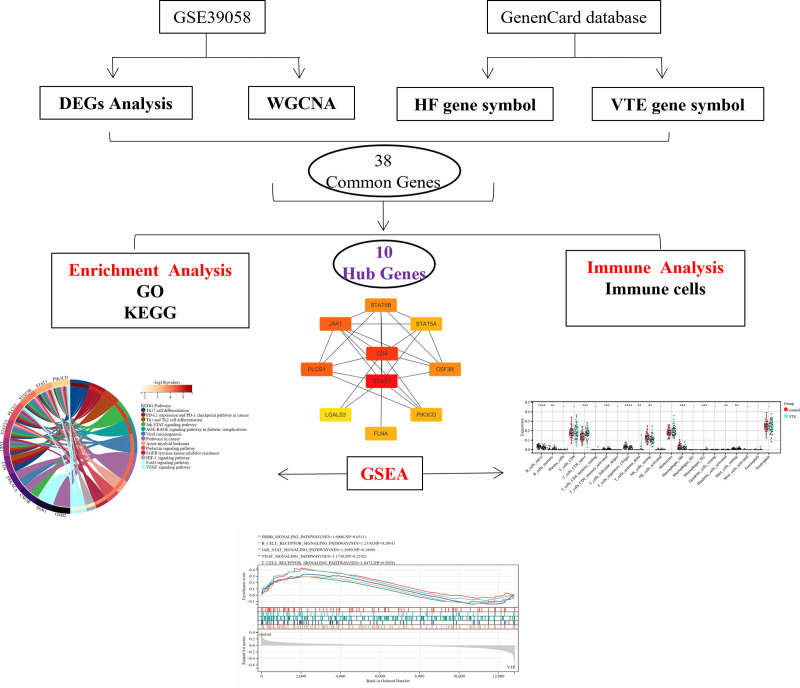
This figure shows the flow structure of the entire article. DEG = differential genes, GO = Gene Ontology, GSEA = Gene Set Enrichment Analysis, HF = hip fracture, KEGG = Kyoto Encyclopedia of Genes and Genomes, VTE = venous thromboembolism, WGCNA = weighted gene co-expression network analysis.

**Figure 2.**
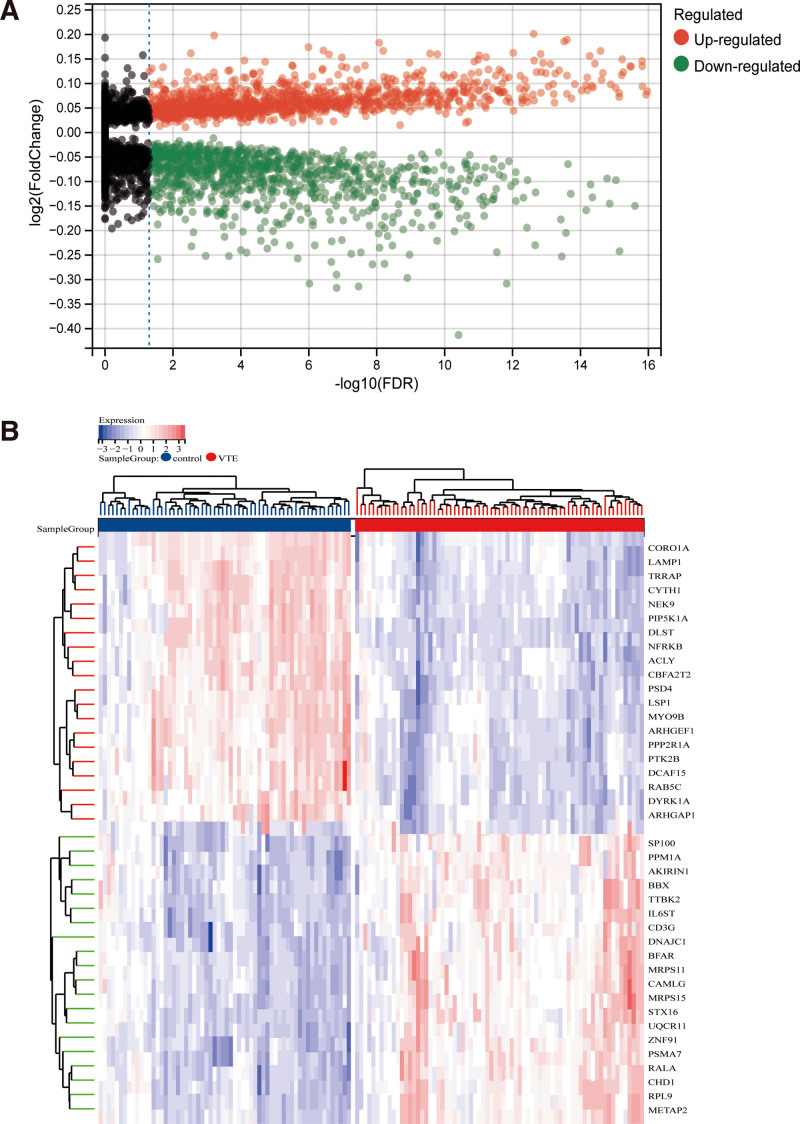
Limma analysis. (A) Differential volcano plot shows differential gene expression in general, with red dots denoting up-regulated genes, green dot shapes denoting down-regulated genes, and gray dots denoting genes with no significant differences. (B) Differential heatmap clearly shows genes with significant differences between groups, with red denoting up-regulation and blue denoting down-regulation.

Figure 1 depicts the bioinformatics analytic procedure used in this investigation. GEO-NCBI provided the data, and GSE19151 concluded 70 DVT and 63 normal patient controls microarray data. An analysis of the variations between the 2 groups revealed that, of the 12,823 genes, 2273 differential genes were found, with 1233 of them being upregulated and 1040 being downregulated (Tables S1 and S2, Supplemental Digital Content, http://links.lww.com/MD/O240; Fig. 2A, B).

### 3.2. Results of WGCNA

**Figure 3.**
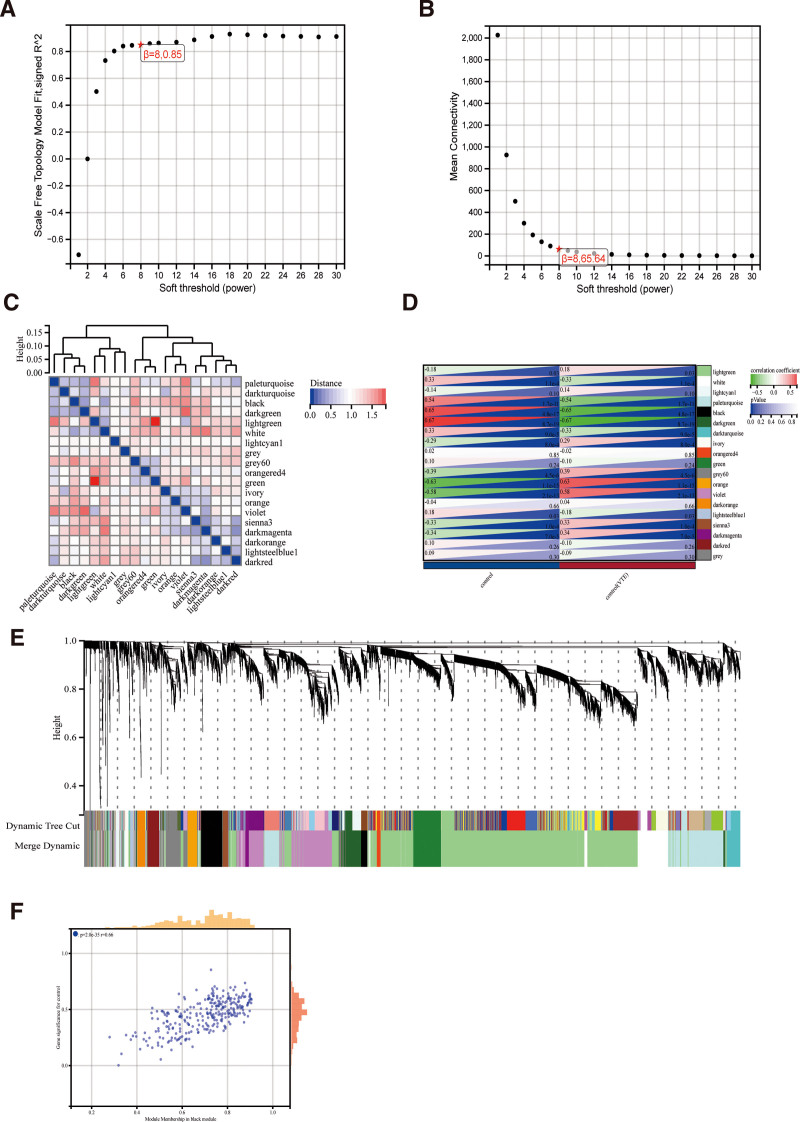
Results of WGCNA. (A) Corresponding scale-free topological models fitted with fit indices at different soft-threshold powers. (B) Corresponding average connectivity values at different soft-threshold powers. (C) Module eigenvector clustering analysis of modules. (D) Correlation analysis of modules with clinical features. (E) Cluster dendrograms of genes. (F) Correlation analysis between MM and GS. WGCNA = weighted gene co-expression network analysis. MM = Module Membership, GS = Gene Significance, WGCNA = weighted gene co-expression network analysis.

Weighted gene co-expression networks were created using the expression profiles of 12,823 genes that were taken from GSE19151 after the aberrant samples were eliminated and the genes were screened. The average connection value was 65.64 and the scale independence was 0.85 when the soft threshold power was set at 8 (Fig. 3A, B). By setting the cut height to 0.25 and the minimum module size to 30, dynamic tree cutting produced 19 distinct co-expression modules (Fig. 3E). A feature of the module Each module underwent vector clustering analysis, which revealed that the lightgreen and green modules had the greatest distance (Fig. 3C). A correlation study between each module and clinical characteristics was then carried out. DVT showed a negative association with the green module (correlation coefficient = −0.1, *P* = .24; Fig. 3D) and a positive correlation with the lightgreen module (correlation coefficient = 0.18, *P* = .03). Additionally, Module Membership and Gene Significance correlation analysis revealed a strong association between these genes and both modules and phenotypes (*R* = 0.66, *P* = 2.0e−35; Fig. 3F). After extracting every module gene, we were left with 1850 module genes (Table S3, Supplemental Digital Content, http://links.lww.com/MD/O240).

### 3.3. Deep vein thrombosis biomarker identification

**Figure 4.**
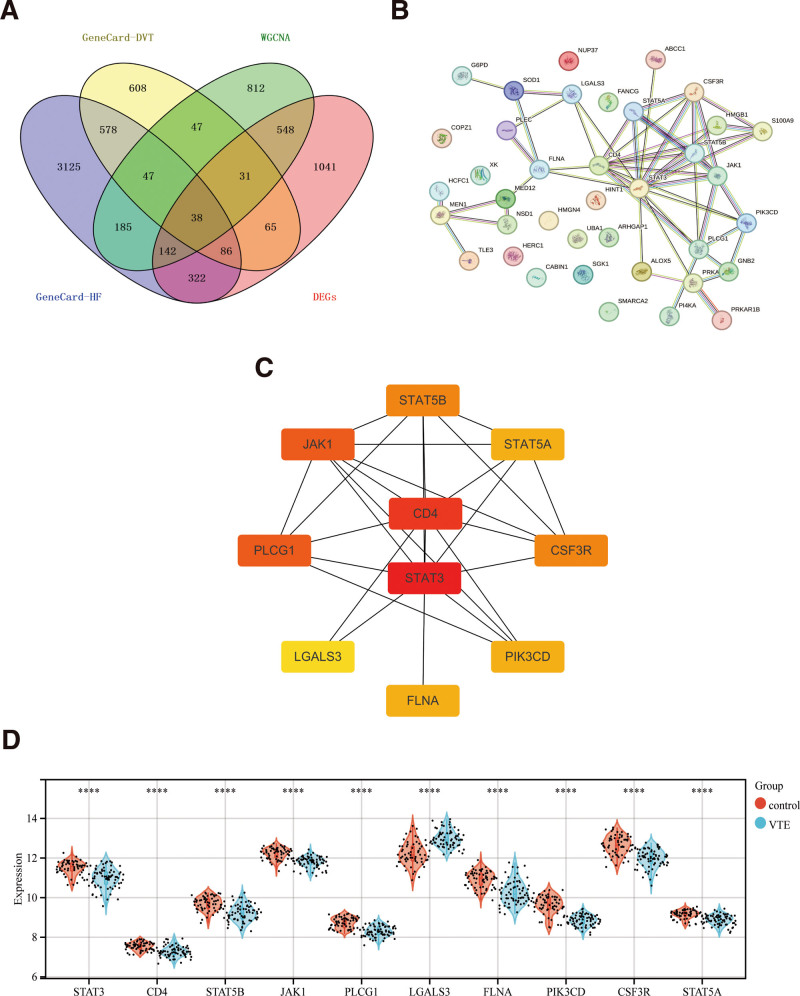
(A) Veen plot of 38 significant intersecting gene sets; (B) 38 key gene sets obtained after PPI screening; (C) 10 pivotal gene sets obtained after PPI screening; (D) differential gene boxplot of pivotal genes showing differential expression statistics (*** *P* < .001, ** *P* < .01, * *P* < .005). PPI = Protein-Protein interaction

Relevant searches were performed in GeneCards Disease Database to obtain 4523 targets of action for hip fracture disease and 1500 targets of action for venous thrombosis to take intersections with 1850 modular genes and 2273 differential genes, respectively, and ultimately 38 significant common targets sets were obtained (Tables S4–S6, Supplemental Digital Content, http://links.lww.com/MD/O240; Fig. 4A). A set of 38 key genes and 10 important hub genes were obtained by PPI and Cytoscape screening (Fig. 4B, C). We examined the expression of ten key genes after comparing them with the raw expression difference data, and the statistics of the pivotal genes’ differential expression are displayed in the differential gene box-and-line plot (Fig. 4D), of which the statistically significant pivotal genes were STAT3, CD4, STAT5B, JAK1, PLCG1, LGALS3, FLNA, PIK3CD, CSF3R, and STAT5A.

### 3.4. Results of bioprocess analyses

**Figure 5.**
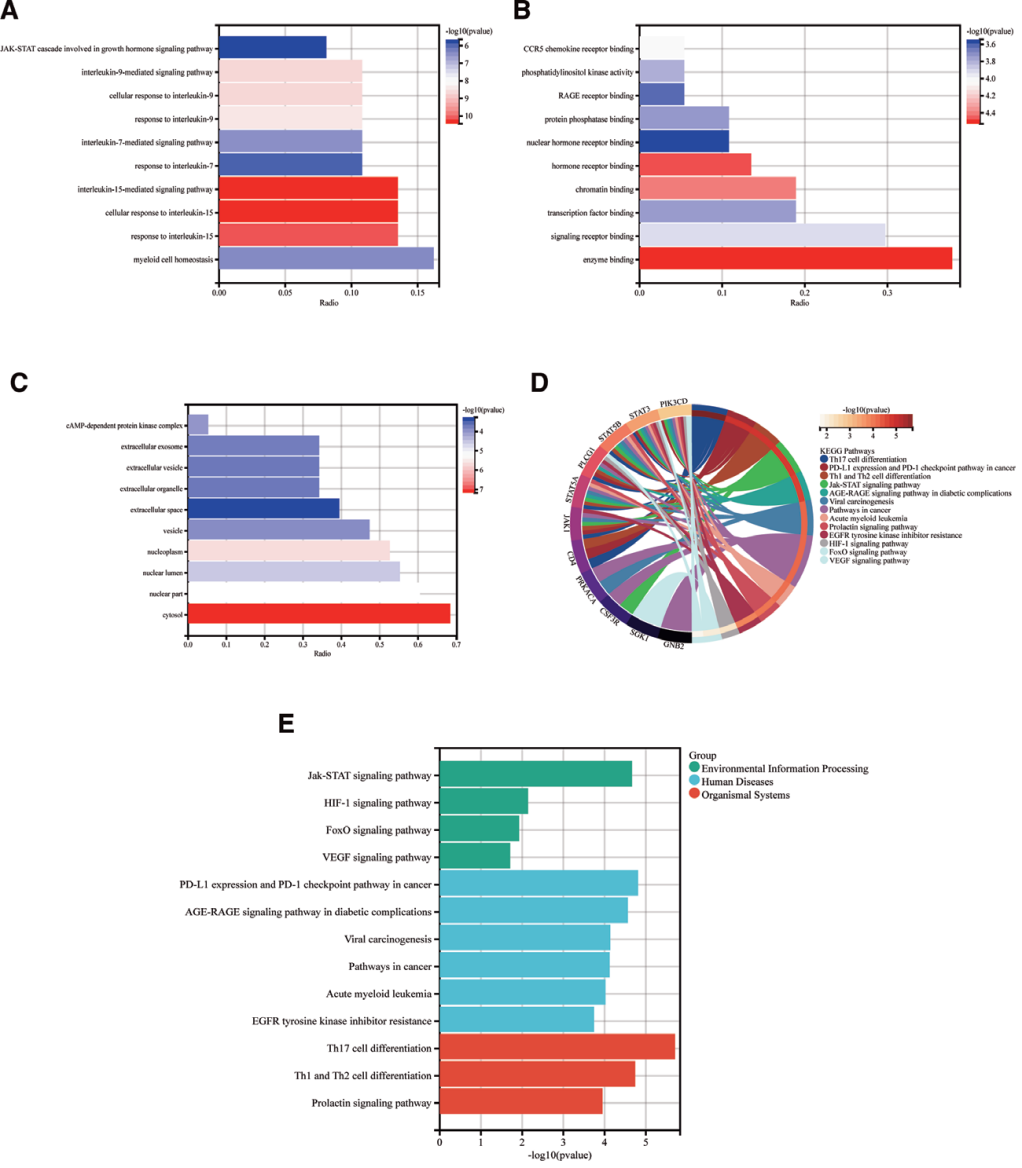
(A) Results of BP enrichment analysis based on hub genes. (B) Results of CC enrichment analysis based on hub genes. (C) Results of MF enrichment analysis based on hub genes. (D) Circle plot analysis of KEGG enrichment based on hub genes. (D) Bar graph analysis of KEGG enrichment based on hub genes. BP = biological process, CC = cellular component, KEGG = Kyoto Encyclopedia of Genes and Genomes, MF = molecular function.

A sum of 38 intersecting genes were subjected to enrichment analysis in order to investigate the pathophysiology of venous thrombosis. GO enrichment study revealed that the hub genes were enriched to 1581 GO entries, of which 303 were for molecular function (MF), 278 were for cellular component (CC), and 1000 were for biological process (BP; Table S7, Supplemental Digital Content, http://links.lww.com/MD/O240). The top ten enrichment results in BP, CC, and MF are displayed as bar graphs among them (Fig. 5A–C), and the findings demonstrate that GO analysis during DVT mostly involves the JAK-STAT cascade, which is engaged in the growth hormone signaling route, the extracellular exosome, the extracellular vesicle, the extracellular organelle, and the interleukin-9 and interleukin-7 driven signaling pathways. According to KEGG pathway analysis, 173 signaling pathways are expressed during the DVT process (Table S8, Supplemental Digital Content, http://links.lww.com/MD/O240), Furthermore, screening revealed that these pathways were primarily implicated in the VEGF signaling route, FoxO signaling pathway, HIF-1 signaling pathway, and Jak-STAT signaling pathway (Fig. 5D). DVT may have a close relationship with the immune system in humans, namely with regard to the differentiation of Th1, Th2, and Th17 immune cells (Fig. 5E).

### 3.5. Examining the findings of immunological infiltration

**Figure 6.**
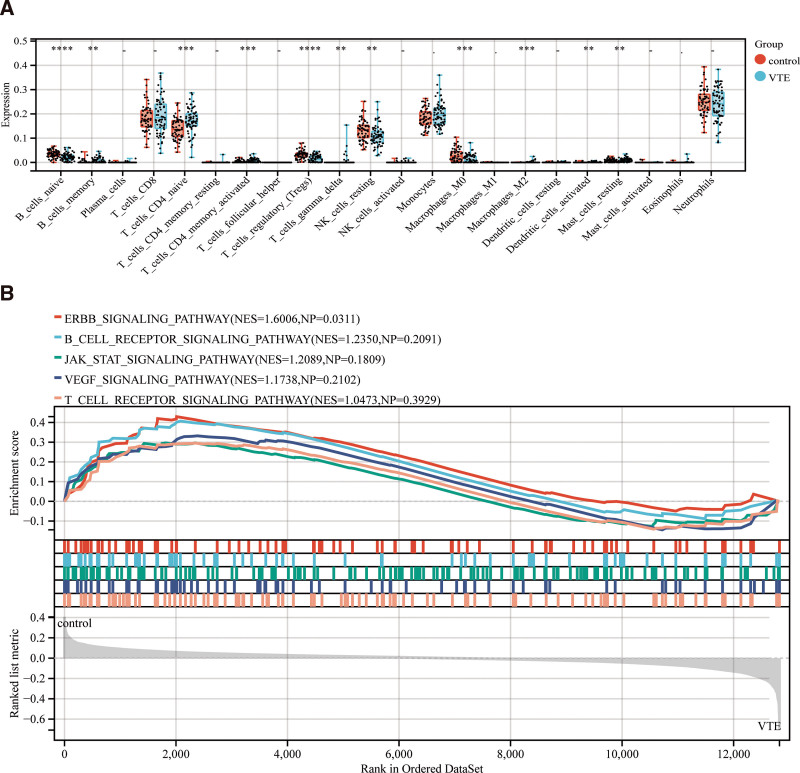
(A) Statistical significance of intergroup differences in immune-infiltrating cells for hub genes. (B) Gene Set Enrichment Analysis results.

Using the CIBERSORT algorithm, we were able to determine the immune infiltration score of GSE19151. The results of the immune infiltration score are displayed in a difference box line plot (Table S9, Supplemental Digital Content, http://links.lww.com/MD/O240, Fig. 6A). The findings indicate significant differences between B cells naïve, B cells memory, T cells CD4 naïve, T cells CD4 memory activated, T cells regulatory (Tregs), T cells gamma-delta, NK cells resting, Macrophages M0, Macrophages M2, Dendritic cells activated, and Mast cells resting (** *P* < .01; Fig. 6B). In addition, further Gene Set Enrichment Analysis validation again demonstrated that JAK_STAT_SIGNALIN, VEGF_SIGNALING_PATHWAY, In the formation of DVT, B_CELL_RECEPTOR_SIGNALING_PATHWAY and T_CELL_RECEPTOR_SIGNALING_PATHWAY are crucial.

## 4. Discussion

HF is the leading cause of fractures in people 65 years and older and are among the top ten reasons why older persons lose disability-adjusted life years. Worldwide, it is anticipated that HF may increase significantly as a result of population aging and other factors.^[25–27]^ For these patients, DVT is a frequent consequence and a leading cause of morbidity and death.^[28]^ Previously, it was thought that thrombosis and the inflammatory immune response were 2 separate pathophysiological processes. However, as the study progressed, it was discovered that thrombosis and the inflammatory immune response were partially cross-linked and influenced one another in molecular components and signaling pathways. This, in part, supports the idea that the inflammatory immune response and VTE are related, and it offers a theoretical foundation for investigating the relationship between immune-inflammation and the risk of VTE in older adults who have had hip fractures.^[3,29]^ Nevertheless, little is known about the precise function of immune-inflammatory pathways in DVT. Thus, in order to guide the clinical care of hip fractures in the elderly, we will uncover the mechanism of immune inflammation’s function in DVT in this study.

**Figure 7.**
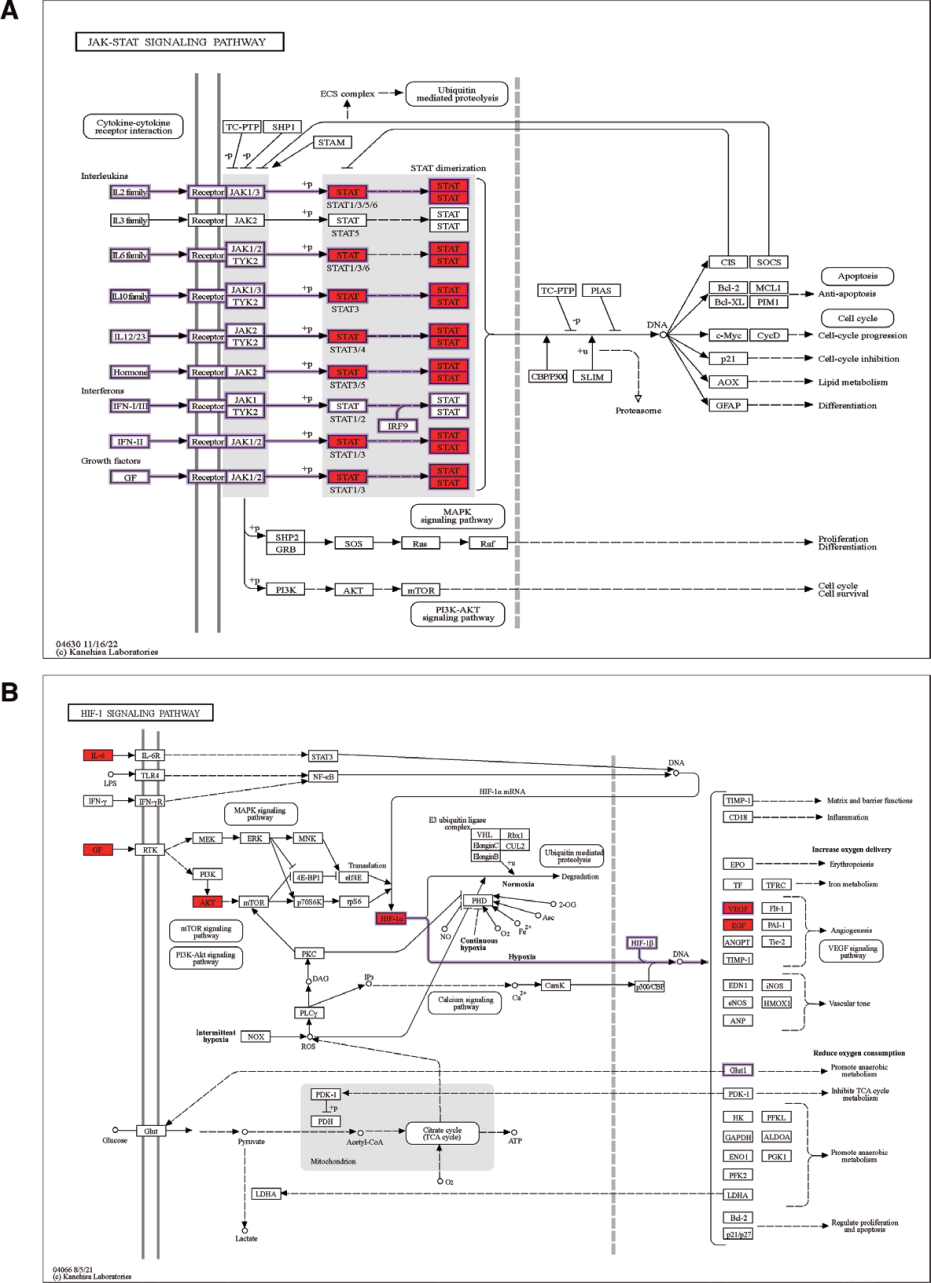
(A) Jak-STAT signaling pathway and related target expression, such as STAT. (B) HIF signaling pathway and related target expression, such as IL6, HIF-1α, EGF, VEGF. EGF = epidermal growth factor, HIF = hypoxia-inducible factor, IL = interleukin, STAT = signal transducer and activator of transcription, VEGF = vascular endothelial growth factor.

In our investigation, we initially used the GEO database to examine gene expression in VTE patients and healthy persons, obtaining 2273 overexpressed genes and 1850 modular genes. Then, we further analyzed the mechanism of DVT in HF by combining the data of venous thrombosis with hip fracture. After 38 effector genes were first screened, 10 crucial genes linked to immunological inflammation were found using PPI network, GO, and KEGG enrichment analyses, that is, STAT3, CD4, STAT5B, JAK1, PLCG1, LGALS3, FLNA, PIK3CD, CSF3R, and STAT5A. Screening showed that VEGF, FoxO, Hif-1, and Jak-STAT signaling pathways were strongly associated with DVT. Constitutive activation of signal transducer and activator of transcription family members has been directly linked to tumor angiogenesis and other cancer progression pathways, according to numerous studies. The stimulation of pro-angiogenic factors in response to hypoxia and pro-inflammatory cytokines is specifically tightly linked to STAT3, whereas STAT5 promotes the expression and release of angiogenic factors.^[30]^ One of the main cytokine signaling mechanisms is the Jak-STAT system. The phosphorylation of JAK2 and its downstream components, STAT1 and STAT3, was shown to be strongly inhibited by the selective JAK2 inhibitor AG490. AG490 dramatically decreased the concentration of macrophages in the kidney and inhibited the production of the MCP-1 and ICAM-1 proteins. Additionally, using AG490 right after ischemia greatly reduced kidney damage.^[31]^ These studies and the current experiment’s findings led us to hypothesize that the Jak-STAT signaling pathway, which is mediated by the STAT family, is crucial to the development of DVT and that preventing the development of DVT following a hip fracture can be achieved by modifying related targets and pathways (Fig. 7A). In a study, found that inhibition of JAK-STAT signaling with the clinically available JAK 2 inhibitor, ruxolitinib, abolished myeloproliferative tumor formation and reduced thrombosis in a mouse model of deep vein stenosis. This provides theoretical support for the application of STAT biomarkers in clinical practice.^[32]^ Every cell in vertebrate beings receives oxygen and nutrients from the circulatory system. One important modulator of the hypoxic/ischemic vascular response that promotes involvement in vascular angiogenesis is hypoxia-inducible factor 1 (HIF-1). Through the transcriptional activity of HIF-1, hypoxia triggers the development of important angiogenic growth factors, such as platelet-derived growth factor B (PDGFB), vascular endothelial growth factor (VEGF), stromal-derived factor 1 (SDF1), angiopoietin 2 (ANGPT2), and placental growth factor (PGF).^[33]^ Based on available data, the NF-κB pathway is primarily responsible for the hypoxia-mediated regulation of angiogenic genes linked to inflammation.^[34]^ Through its target genes, such as VEGF, which influences endothelial cell permeability, and PAI-1, which slows thrombo fibrinolysis, HIF-1α can accelerate the start or progression of venous thrombosis. Upregulation of HIF-1α in endothelial cells and myeloid-erived phagocytes (e.g., monocytes and macrophages) can also stimulate proinflammatory processes via mediators such as TNF-α, IL-1, and NO, leading to venous thrombosis.^[35]^ In conclusion, the HIF pathway contributes to the regulation of physiological and pathological vessel wall remodeling (Fig. 7B). Resveratrol may help rats with DVT by blocking the HIF-1α/NLRP3 pathway, according to an experimental investigation.^[36]^ Additionally, EGCG and warfarin altered HIF-1α and VEGF to prevent inflammation and rabbit DVT via the PI3K/AKT and ERK1/2 signaling pathways, according to another study.^[37]^ Therefore, we believe that HIF-1α and VEGF are significant as predictive markers of thrombosis in the clinic. To sum up, we think that these markers could be used as DVT predictors, and we want to progressively carry out pertinent clinical research and evaluate the importance of pertinent indicators.

It is now commonly acknowledged that inflammation and immune system response play a significant role in the development and progression of DVT. Based on 38 intersecting genes, immune infiltration results were obtained by the CIBERSORT algorithm. Further investigation revealed that patients with DVT have high levels of immune cell infiltration, including T cells_CD4_naïve, T cells_memory_activated, T cells_regulatory_(Tregs), B cells_naïve, B cells_memory, Macrophages_M0, and Macrophages_M2. The development of helper Th17 cells, which release cytokines like IL-17 and IL-22, among others, depends on STAT3, a signal that comes after IL-6. STAT5A and STAT5B are the 2 STAT5 genes. A downstream signal for numerous cytokines, STAT5B is essential for the development of immune cells and the immunological response, particularly for T and NK cells.^[38]^ Furthermore, through downregulating CD4 and CD8 T cells and encouraging the development of regulatory T cells, HIF inhibits adaptive immune responses. In light of this, endothelial cells and immune cells have a strong communication line during inflammation. Some immune cells, such as neutrophils and monocytes, are crucial for inflammation-associated angiogenesis and are also important sources of pro-angiogenic factors, such as VEGFA, FGF2, and MMP9, as well as STAT3-mediated signaling.^[35]^ Eosinophil activation results in the degranulation of pro-angiogenic factors in vitro, and lymphocytes have been demonstrated to either directly or indirectly control angiogenesis; for example, B cells release angiogenic factors that resemble those released by myeloid cells. In a similar manner, regulatory T cells block effector Th1 cells that release IFN-γ by releasing VEGFA, which promotes angiogenesis. Th1 and cytotoxic T cells release interferon-gamma (IFN-γ), which prevents tumor angiogenesis by restricting the growth of endothelial cells, while helper T cell 2 (Th2) encourages the differentiation of tumor-infiltrating monocytes and macrophages into pro-angiogenic tumor-associated macrophages (TAMs).^[39]^ Experimentally, Similar to wound healing, thrombus regression begins with neutrophil-driven effects and progresses to fibrinolysis, neovascularization, and collagen replacement mediated by monocytes and macrophages. Monocyte-derived macrophages are the main effectors of immune-directed DVT regression. In the case of patients with pathological DVT, polarized, exists in proinflammatory macrophages sampled from blood. These proinflammatory human macrophages are characterized by increased IL-6, TNF-a, and cell adhesion markers. Multiple other markers of proinflammatory macrophages are described, and they differ between humans and mice.^[40]^

Although the regulation of angiogenesis by these immune cells has been variously investigated in tumors, we hypothesize that these molecular mechanisms are equally applicable to DVT formation after hip fracture. Despite the fact that this study identified these indicators and some immune cell functions, the bioinformatics-based results are lacking. First, the study’s sample size and data volume are tiny; second, the indications require additional verification. In addition, the markers are located here. As a result, in later research, we conducted both human and animal validation using a wider range of samples.

## 5. Conclusion

In summary, we established a hip fracture complicating DVT disease prediction model based on 38 intersecting genes, and we found that the pathogenesis of DVT was mainly associated with the abnormal expression of Jak-STAT signaling pathway, HIF-1 signaling pathway, FoxO signaling pathway, and VEGF signaling pathway. In addition, immune-inflammatory responses regulated by B cells, T cells, and macrophages play an important role in venous thrombosis. We finally confirmed that the pivotal genes STAT3, CD4, STAT5B, JAK1, and STAT5A can be applied to the diagnosis of DVT, which is beneficial for the prevention and treatment of hip fracture complicating DVT.

## Author contributions

**Conceptualization:** Fei Liu, Qing Shang, Zongchao Liu.

**Formal analysis:** Yongliang Mei.

**Funding acquisition:** Zongchao Liu.

**Methodology:** Yongliang Mei, Jingwen Chen.

**Software:** Yang Zhou.

**Visualization:** Daqian Zhou.

**Writing – original draft:** Zhijiang Fu, Chao Song.

**Writing – review & editing:** Fei Liu, Qing Shang.

## Supplementary Material



## References

[1] Taoka T Ohmori T Kanazawa T Toda K Ishihara T Ito Y. Delayed surgery after hip fracture affects the incidence of venous thromboembolism. J Orthop Surg Res. 2023;18:630. 37641109 10.1186/s13018-023-04122-8 PMC10463883

[2] Trivedi NN Sivasundaram L Wang C. Chemoprophylaxis for the hip fracture patient: a comparison of warfarin and low-molecular-weight heparin. J Orthop Trauma. 2019;33:216–9. 31008818 10.1097/BOT.0000000000001435

[3] Navarrete S Solar C Tapia R Pereira J Fuentes E Palomo I. Pathophysiology of deep vein thrombosis. Clin Exp Med. 2023;23:645–54. 35471714 10.1007/s10238-022-00829-w

[4] Pincus D Ravi B Wasserstein D. Association between wait time and 30-day mortality in adults undergoing hip fracture surgery. JAMA. 2017;318:1994–2003. 29183076 10.1001/jama.2017.17606 PMC5820694

[5] Peng J Wang H Zhang L Lin Z. Construction and efficiency analysis of prediction model for venous thromboembolism risk in the elderly after hip fracture. Zhong Nan Da Xue Xue Bao Yi Xue Ban. 2021;46:142–8. 33678650 10.11817/j.issn.1672-7347.2021.190722 PMC10929787

[6] Sheehan KJ Sobolev B Guy P; Canadian Collaborative Study on Hip Fractures. Feasibility of administrative data for studying complications after hip fracture surgery. BMJ Open. 2017;7:e015368. 10.1136/bmjopen-2016-015368 PMC5623359 28473519

[7] Protty MB Aithal S Hickey B Pettit R Johansen A. Mechanical prophylaxis after hip fracture: what is the risk of deep vein thrombosis? A retrospective observational study. BMJ Open. 2015;5:e006956. 10.1136/bmjopen-2014-006956 PMC4330328 25678543

[8] Zhuang Q He Q Aikebaier A Chen W Liu J Wang D. The risk factors for new-onset calf muscle venous thrombosis after hip fracture surgery. J Pers Med. 2023;13:257. 36836491 10.3390/jpm13020257 PMC9964475

[9] Colling ME Tourdot BE Kanthi Y. Inflammation, infection and venous thromboembolism. Circ Res. 2021;128:2017–36. 34110909 10.1161/CIRCRESAHA.121.318225 PMC8202069

[10] Mereweather LJ Constantinescu-Bercu A Crawley JTB Salles C II. Platelet-neutrophil crosstalk in thrombosis. Int J Mol Sci . 2023;24:1266. 36674781 10.3390/ijms24021266 PMC9861587

[11] Martinod K Deppermann C. Immunothrombosis and thromboinflammation in host defense and disease. Platelets. 2021;32:314–24. 32896192 10.1080/09537104.2020.1817360

[12] Budnik I Brill A. Immune factors in deep vein thrombosis initiation. Trends Immunol. 2018;39:610–23. 29776849 10.1016/j.it.2018.04.010 PMC6065414

[13] Wang G Zhao W Yang Y Yang G Wei Z Guo J. Identification of biomarkers of venous thromboembolism by bioinformatics analyses. Medicine (Baltim). 2018;97:e0152. 10.1097/MD.0000000000010152 PMC5902267 29620629

[14] Shen W Song Z Zhong X. Sangerbox: A comprehensive, interaction-friendly clinical bioinformatics analysis platform. iMeta. 2022;1:e36. 38868713 10.1002/imt2.36 PMC10989974

[15] Feng S Xu Y Dai Z Yin H Zhang K Shen Y. Integrative analysis from multicenter studies identifies a WGCNA-derived cancer-associated fibroblast signature for ovarian cancer. Front Immunol. 2022;13:951582. 35874760 10.3389/fimmu.2022.951582 PMC9304893

[16] Safran M Dalah I Alexander J. GeneCards Version 3: the human gene integrator. Database (Oxford). 2010;2010:baq020. 20689021 10.1093/database/baq020 PMC2938269

[17] Piñero J Ramírez-Anguita JM Saüch-Pitarch J. The DisGeNET knowledge platform for disease genomics: 2019 update. Nucleic Acids Res. 2020;48:D845–55. 31680165 10.1093/nar/gkz1021 PMC7145631

[18] Szklarczyk D Gable AL Nastou KC. The STRING database in 2021: customizable protein-protein networks, and functional characterization of user-uploaded gene/measurement sets. Nucleic Acids Res. 2021;49:D605–12. 33237311 10.1093/nar/gkaa1074 PMC7779004

[19] Otasek D Morris JH Bouças J Pico AR Demchak B. Cytoscape Automation: empowering workflow-based network analysis. Genome Biol. 2019;20:185. 31477170 10.1186/s13059-019-1758-4 PMC6717989

[20] Oda Y Nagasu T Chait BT. Enrichment analysis of phosphorylated proteins as a tool for probing the phosphoproteome. Nat Biotechnol. 2001;19:379–82. 11283599 10.1038/86783

[21] Kanehisa M Furumichi M Tanabe M Sato Y Morishima K. KEGG: new perspectives on genomes, pathways, diseases and drugs. Nucleic Acids Res. 2017;45:D353–61. 27899662 10.1093/nar/gkw1092 PMC5210567

[22] Lei X Lei Y Li JK. Immune cells within the tumor microenvironment: Biological functions and roles in cancer immunotherapy. Cancer Lett. 2020;470:126–33. 31730903 10.1016/j.canlet.2019.11.009

[23] Craven KE Gökmen-Polar Y Badve SS. CIBERSORT analysis of TCGA and METABRIC identifies subgroups with better outcomes in triple negative breast cancer. Sci Rep. 2021;11:4691. 33633150 10.1038/s41598-021-83913-7 PMC7907367

[24] Subramanian A Tamayo P Mootha VK. Gene set enrichment analysis: a knowledge-based approach for interpreting genome-wide expression profiles. Proc Natl Acad Sci USA. 2005;102:15545–50. 16199517 10.1073/pnas.0506580102 PMC1239896

[25] McDonough CM Harris-Hayes M Kristensen MT. Physical therapy management of older adults with hip fracture. J Orthop Sports Phys Ther. 2021;51:CPG1–CPG81. 10.2519/jospt.2021.0301 33522384

[26] Sing CW Lin TC Bartholomew S. Global epidemiology of hip fractures: secular trends in incidence rate, post-fracture treatment, and all-cause mortality. J Bone Miner Res. 2023;38:1064–75. 37118993 10.1002/jbmr.4821

[27] Buzkova P Cauley JA Fink HA Robbins JA Mukamal KJ Barzilay JI. Age-related factors associated with the risk of hip fracture. Endocr Pract. 2023;29:478–83. 36889582 10.1016/j.eprac.2023.03.001 PMC10258141

[28] Bengoa F Vicencio G Schweitzer D Lira MJ Zamora T Klaber I. High prevalence of deep vein thrombosis in elderly hip fracture patients with delayed hospital admission. Eur J Trauma Emerg Surg. 2020;46:913–7. 30523360 10.1007/s00068-018-1059-8

[29] Amen TB Varady NH Hayden BL Chen AF. Pathologic versus native hip fractures: comparing 30-day mortality and short-term complication profiles. J Arthroplasty. 2020;35:1194–9. 31987688 10.1016/j.arth.2020.01.003

[30] Cavazzoni A Digiacomo G Volta F. PD-L1 overexpression induces STAT signaling and promotes the secretion of pro-angiogenic cytokines in non-small cell lung cancer (NSCLC). Lung Cancer. 2024;187:107438. 38100954 10.1016/j.lungcan.2023.107438

[31] Yang N Luo M Li R. Blockage of JAK/STAT signalling attenuates renal ischaemia-reperfusion injury in rat. Nephrol Dial Transplant. 2008;23:91–100. 17670769 10.1093/ndt/gfm509

[32] Wolach O Sellar RS Martinod K. Increased neutrophil extracellular trap formation promotes thrombosis in myeloproliferative neoplasms. Sci Transl Med. 2018;10:eaan8292. 29643232 10.1126/scitranslmed.aan8292 PMC6442466

[33] Rey S Semenza GL. Hypoxia-inducible factor-1-dependent mechanisms of vascularization and vascular remodelling. Cardiovasc Res. 2010;86:236–42. 20164116 10.1093/cvr/cvq045 PMC2856192

[34] Oliver KM Taylor CT Cummins EP. Hypoxia. Regulation of NFkappaB signalling during inflammation: the role of hydroxylases. Arthritis Res Ther. 2009;11:215. 19291263 10.1186/ar2575 PMC2688226

[35] Lim CS Kiriakidis S Sandison A Paleolog EM Davies AH. Hypoxia-inducible factor pathway and diseases of the vascular wall. J Vasc Surg. 2013;58:219–30. 23643279 10.1016/j.jvs.2013.02.240

[36] Fei J Qin X Ma H. Resveratrol ameliorates deep vein thrombosis-induced inflammatory response through inhibiting HIF-1α/NLRP3 pathway. Inflammation. 2022;45:2268–79. 35655037 10.1007/s10753-022-01689-y

[37] Li Y Ge JP Ma K Yin YY He J Gu JP. The combination of EGCG with warfarin reduces deep vein thrombosis in rabbits through modulating HIF-1α and VEGF via the PI3K/AKT and ERK1/2 signaling pathways. Chin J Nat Med. 2022;20:679–90. 36162953 10.1016/S1875-5364(22)60172-9

[38] Banerjee S Biehl A Gadina M Hasni S Schwartz DM. JAK-STAT signaling as a target for inflammatory and autoimmune diseases: current and future prospects. Drugs. 2017;77:521–46. 28255960 10.1007/s40265-017-0701-9 PMC7102286

[39] Huang Y Kim BYS Chan CK Hahn SM Weissman IL Jiang W. Improving immune-vascular crosstalk for cancer immunotherapy. Nat Rev Immunol. 2018;18:195–203. 29332937 10.1038/nri.2017.145 PMC5922422

[40] Henke PK Nicklas JM Obi A. Immune cell-mediated venous thrombus resolution. Res Pract Thromb Haemost. 2023;7:102268. 38193054 10.1016/j.rpth.2023.102268 PMC10772895

